# Effortless Attention as a Biomarker for Experienced Mindfulness Practitioners

**DOI:** 10.1371/journal.pone.0138561

**Published:** 2015-10-12

**Authors:** Guaraci Ken Tanaka, Tolou Maslahati, Mariana Gongora, Juliana Bittencourt, Luiz Carlos Serramo Lopez, Marcelo Marcos Piva Demarzo, Henning Budde, Silmar Teixeira, Luis Fernando Basile, Javier Garcia Campayo, Mauricio Cagy, Pedro Ribeiro, Bruna Velasques

**Affiliations:** 1 Brain Mapping and Sensory Motor Integration, Institute of Psychiatry of the Federal University of Rio de Janeiro (IPUB/UFRJ), Rio de Janeiro, Brasil; 2 Institute of Applied Neuroscience (INA), Rio de Janeiro,Brasil; 3 Mente Aberta, Brazilian Center for Mindfulness and Health Promotion, Federal University of Sao Paulo (UNIFESP), São Paulo, Brasil; 4 Brain Mapping and Plasticity Laboratory, Federal University of Piauí (UFPI), Piaui, Brasil; 5 Laboratory of Psychophysiology, Faculdade da Saúde, UMESP, São Paulo, Brazil; 6 Migue Servet University Hospital, Aragonese Institute of Health Sciences, University of Zaragoza, Zaragoza, Spain; 7 Bioscience Department, School of Physical Education of the Federal University of Rio de Janeiro (UFRJ), Rio de Janeiro, Brasil; 8 Biomedical Engineering Program, COPPE, Federal University of Rio de Janeiro, Rio de Janeiro, Brasil; 9 Neurophysiology and Neuropsychology of Attention, Institute of Psychiatry of the Federal University of Rio de Janeiro (IPUB/UFRJ), Rio de Janeiro, Brasil; 10 Faculty of Human Sciences, Medical School Hamburg, Hamburg, Germany; 11 Sport Science, Reykjavik University, Reykjavik, Iceland; 12 Behavioral Ecology and Psychobiology Laboratory, Department of Systematics and Ecology, Universidade Federal da Paraiba (UFPB), Paraíba, Brasil; 13 Veiga de Almeida University, Rio de Janeiro, Brasil; 14 Division of Neurosurgery, Department of Neurology, University of São Paulo Medical School, São Paulo, Brasil; Osaka University Graduate School of Medicine, JAPAN

## Abstract

**Objective:**

The present study aimed at comparing frontal beta power between long-term (LTM) and first-time meditators (FTM), before, during and after a meditation session. We hypothesized that LTM would present lower beta power than FTM due to lower effort of attention and awareness.

**Methods:**

Twenty one participants were recruited, eleven of whom were long-term meditators. The subjects were asked to rest for 4 minutes before and after open monitoring (OM) meditation (40 minutes).

**Results:**

The two-way ANOVA revealed an interaction between the group and moment factors for the Fp1 (p<0.01), F7 (p = 0.01), F3 (p<0.01), Fz (p<0.01), F4 (p<0.01), F8 (p<0.01) electrodes.

**Conclusion:**

We found low power frontal beta activity for LTM during the task and this may be associated with the fact that OM is related to bottom-up pathways that are not present in FTM.

**Significance:**

We hypothesized that the frontal beta power pattern may be a biomarker for LTM. It may also be related to improving an attentive state and to the efficiency of cognitive functions, as well as to the long-term experience with meditation (i.e., life-time experience and frequency of practice).

## Introduction

Many studies seek to understand the neurophysiology of sustained attention [1–5]. In conditions when more external stimuli than can be fully processed activate the central nervous system (CNS), attentional impairments result, such as stress, distraction, forgetfulness, anxiety, memory loss, and fatigue [6–8]. This impairment is discussed in studies of working memory, default mode network, and attentional disorder studies. Recent studies have demonstrated the role of meditation as an important tool for attention management [9–11].

In this context, mindfulness meditation is one important method that increases attention performance. Specially, some studies demonstrated that a specific type of mindfulness, Open Monitoring (OM) [12], increases posterior alpha power with a high frontal theta power [13,14]. Researchers also observed that experienced OM meditators showed an increase of occipital and frontoparietal gamma activity, due to an improvement of sensory awareness [15,16]. Beta activity in relation to meditation has only been investigated in a few studies until now. In the current literature we have found four studies that examined beta power and meditation. One study observed the general increase of beta power in the elderly meditation novices [17]. Dor-Ziderman et al. (2013) investigated the long-term OM and observed a beta power decrease in the ventral medial prefrontal cortex [17,18]. Further studies showed a different pattern of frontal beta power when compared to another meditation practice [19,20].

OM meditation favors sustained attention without judgment of ongoing phenomena [12]. These mindfulness properties are thought to improve self-regulation and stress management by allowing individuals to refrain from trying to control the content of mind [21–23]. Nevertheless, we suggest that frequency plays an important role when comparing long-term meditators (LTM) with first-time meditators (FTM), particularly regarding activity in the frontal areas [20,21,24]. Beta band frequency (13-30Hz) is associated with attention, vigilance and processing information [25,26], but the literature regarding the neurophysiology of frontal beta activity and meditation is scant. We observed a lack of consistent reports on the regulatory mechanisms of beta oscillations related to the self-awareness process in OM. In this context, the aim of the present study was to investigate frontal beta power differences between long-term and first-time meditators, before, during and after a meditation session. We expected increased beta for both groups during OM versus during rest, before and after OM, but generally lower beta in LTM vs. FTM due to lower sustained attention effort expected in the group of experienced meditators.

## Materials and Methods

### Participants

We recruited twenty-one participants, out of which eleven were experienced meditators (7 men, mean age 43,8 ± 17,53) and ten were healthy first-time meditators (5 men, mean age 40,10 ± 14,72). The group of experienced meditators includes monks and laymen from various Buddhist traditions, and were recruited from three major meditation centers (i.e., Zen, Kadampa and Vipassana), localized around the Federal University of Rio de Janeiro (UFRJ). The group of first-time meditators (i.e., control group) was recruited at the UFRJ. We contacted all the participants two weeks before starting the research. The condition for the experienced meditators was to have been practicing regularly at least for the last five years (12,23 years of practice ± 7,65), while living under the same social conditions as the control group (i.e. in the same city, and to be exposed to the same environment; i.e., no secluded life in Buddhist centers). All participants were medication-free and had no sensory, motor, cognitive or attention deficits that could affect their performance. Subjects gave their written consent (according to the Helsinki Declaration) to participate in the study. The Ethics Committee of the Federal University of Rio de Janeiro (IPUB/UFRJ) approved the experiment.

### Task protocol

Subjects sat in a straight position, in a darkened and noise-free room, to minimize sensory interference. First, we recorded 4 minutes of resting EEG (resting instructions were particularly emphasized for meditators, to refrain them from entering the meditative state). After that the subjects were recorded while performing the OM for 40 minutes. The experiment ended by recording 4 minutes of rest again. An auditory signal marked the beginning and end of each stage.

Mindfulness instructions for LTM and FTM were: “pay attention to whatever comes into your awareness. Whatever it is, a stressful thought, an emotion or body sensation, just let it pass in an effortless way, without trying to maintain it or change it in any way, until something else comes into your consciousness”[27]. The researcher gave the instructions right before the start of the practice, as we believed this would promote better motivation as a first attempt of a new activity. Due to its simplicity, the technique could be implemented by all subjects, and we considered this as particularly important, since subjects reported that they had no problems to follow the instructions.

### EEG recording

The International 10/20 EEG electrode system (Jasper, 1958) was used with a 20-channel EEG system (Braintech-3000, EMSA Medical Instruments, Brazil). The 20 electrodes were arranged on a nylon cap (ElectroCap Inc., Fairfax, VA, USA) yielding mono-polar derivation using the earlobes as reference. The impedance of EEG and EOG electrodes was kept between 5–10 kΩ. The amplitude of the recorded data was less than 70μV. The EEG signal was amplified with a gain of 22.000 Hz, analogically filtered between 0.01Hz (high-pass) and 80Hz (low-pass), and sampled at 200 Hz. The software *Data Acquisition* (Delphi 5.0) from the Brain Mapping and Sensory Motor Integration Lab, was employed with the notch (60 Hz) digital filter.

### Data analysis

Data analysis was performed by using MATLAB 5.3 (Mathworks, Inc.) and EEGLAB toolbox (http://sccn.ucsd.edu/eeglab). We applied a visual inspection and Independent Component Analysis (ICA) to remove possible sources of artifacts produced by the task (i.e., blink, muscle). We collected the data using the bi-auricular reference. Furthermore the data was transformed (re-referenced) to common average reference, after conducting the artifact elimination by ICA filtering.

At first, we divided the tasks (rest 1, meditation, rest 2) into segments of 6000 data points, corresponding to 30-second blocks. Secondly, as meditation trials lasted 40 minutes [28,29,25], we chose to analyze eight blocks (total of 4 min), corresponding to the moment between 30–34 minutes, which is consistent with the moment in which experienced meditators recognize entering a deeper state [26]. For that moment we had 24 trials of 30-second blocks. A fast Fourier transform method was used to obtain the mean power amplitudes in the beta (13–30 Hz) band.

The number of samples was 6000 (30s × 200Hz) with rectangular windowing. We calculated absolute beta power on each lead individually every four seconds, totaling seven excerpts for each block. Thus, 1680 was the total data of the control group (24 trials x 7 absolute power samples x 10 subjects) and 1848 the total data of the meditator group (24 trials x 7 absolute power samples x 11 subjects). In fact, this sampling rate is commonly used in other frequencies, but we focused on the beta band due to a specific analyzis [30,31]. As the data was not normally distributed, a logarithmic transformation was used.

### Statistical analysis

In the statistical analysis, we log10-transformed the EEG absolute power values by the SPSS software (version 15.0) to approximate a normal distribution. We performed a two-way ANOVA and a post hoc test (Bonferroni) to analyze the factor group (LTM x FTM) and moment (rest 1, meditation and rest 2) of absolute beta power for each electrode individually. The effect size was calculated as Cohen's d, i.e., changes' mean divided by changes' SD.

We also performed a t-test for all frontal electrodes (Fp1, Fp2, F3, Fz, F4, F7 and F8) comparing LTM versus FTM groups for each moment (rest 1, meditation and rest 2).

## Results

**Fig 1:**
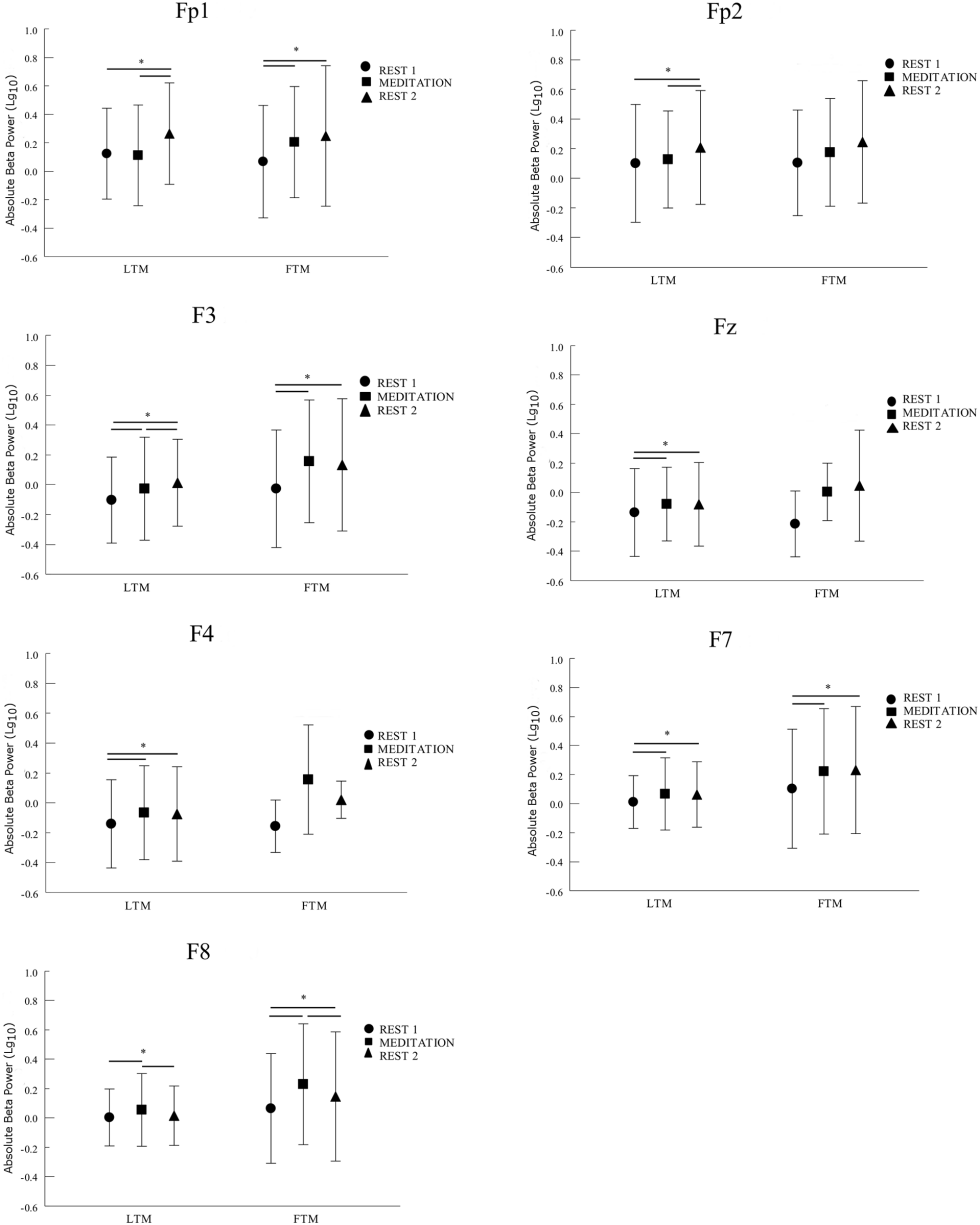
Comparisons of LONG-TERM (LTM) and FIRST-TIME (FTM) meditators in the moment (rest 1 (●); meditation (■); rest 2 (▲)) for all frontal electrodes. Data represent the mean ± SD frequency of logged absolute beta power (log10 transformed) for each electrode. A 2 × 3 ANOVA showed significantly differences in the marked bars (*p* < 0.05; exact values in Table 1). (* represents significant p values) The bars represent the moments that demonstrate difference between them when we examining the interaction.

We analyzed absolute beta power in the frontal area (i.e., Fp1, Fp2, F3, Fz, F4, F7 and F8 electrodes). The two-way ANOVA revealed an interaction between the group and moment factors for the Fp1 (p<0.01), F7 (p = 0.01), F3 (p<0.01), Fz (p<0.01), F4 (p<0.01), F8 (p<0.01) electrodes. Examining the Fp1 interaction, we identified a difference between rest 1 and rest 2; and between meditation and rest 2 for LTM. Specifically, we found a higher beta power for rest 2 when compared to the other moments. We did not find a difference between rest 1 and meditation for LTM. For the FTM group, we observed a difference between rest 1 and meditation; and between rest 1 and rest 2. This group presented lower beta power for rest 1 when compared to the other moments. In other words, beta is increased from rest 1 during meditation for FTM and there is no difference from rest 1 to meditation for LTM (Fig 1 and Table 1). For this electrode, a t-test between groups showed significant difference only for rest 1 and meditation (Table 2). For F3 and Fz, both FTM and LTM had lower beta power at rest 1, when compared to meditation and rest 2 (Fig 1 and Table 1). For these electrodes, a t-test between groups showed significant difference for rest 1, meditation and rest 2 (Table 2). For F4, the LTM and FTM had lower beta power at rest 1 when compared to meditation and rest 2 (Fig 1 and Table 1). As opposed to that, FTM beta power was lower at rest 2, when compared to meditation and higher than rest 1 (Fig 1 and Table 1). For this electrode, a t-test between groups showed significant difference only for meditation and rest 2 (Table 2). For F7, both LTM and FTM had lower beta power at rest 1, when compared to meditation and rest 2 (Fig 1 and Table 1). For this electrode, a t-test between groups showed significant difference for rest 1, meditation and rest 2 (Table 2). Examining the interaction for F8, we found a difference between meditation and rest1, and meditation and rest 2 for LTM. We also identified difference among all the moments for FTM (Table 1). For this electrode, a t-test between groups showed significant difference for rest 1, meditation and rest 2 (Table 2).

**Table 1:** Group x Moment t-test of within groups effects showing p-values and effect size (Cohen’s d) representing differences between two groups. (* represents significant p values).

ELECTRODES (ETA)	GROUP	REST1-MEDIT (Cohen´s)	MEDIT-REST2 (Cohen´s)	REST1-REST2 (Cohen´s)
**Fp1 (0.007)**	LTM	0.33 (-0.05)	<0.01* (-0.25)	<0.01* (-0.22)
	FTM	<0.01* (-0.39)	0.09 (-0.10)	<0.01* (-0.44)
**Fp2 (0.331)**	LTM	0.22 (-0.07)	<0.01* (-0.22)	<0.01* (-0.27)
	FTM	<0.01* (-0.19)	<0.01* (-0.18)	<0.01* (-0.36)
**F3 (0.004)**	LTM	<0.01* (-0.34)	0.02* (0.18)	<0.01* (-0.40)
	FTM	<0.01* (-0.40)	0.33 (0.03)	<0.01* (-0.35)
**F4 (0.028)**	LTM	<0.01* (-0.29)	0.33 (0.18)	<0.01* (-0.21)
	FTM	<0.01* (-0.45)	<0.02* (0.18)	<0.01* (-0.25)
**Fz (0.023)**	LTM	<0.01* (-0.25)	0.33 (0.18)	<0.01* (-0.20)
	FTM	<0.01* (-0.40)	0.01* (0.10)	<0.01* (-0.40)
**F7 (0.002)**	LTM	<0.01* (-0.31)	0.33 (0.06)	<0.01* (-0.15)
	FTM	<0.01* (-0.31)	0.33 (-0.03)	<0.01* (-0.35)
**F8 (0.005)**	LTM	<0.01* (-0.29)	<0.01* (0.20)	0.33 (-0.11)
	FTM	<0.01* (-0.34)	<0.01* (0.16)	<0.01* (-0.16)

**Table 2:** Group x Moment Interaction. T-Test of between groups for each moment, showing p-values and effect size representing differences between two groups. (* represents significant p values).

ELECTRODES	REST 1	MEDITATION	REST 2
**Fp1**	<0.01* (0.14)	<0.01* (-0.23)	0.52 (-0.20)
**Fp2**	0.88 (-0.01)	0.02* (-0.14)	0.11 (-0.09)
**F3**	<0.01* (-0.32)	<0.01* (-0.43)	<0.01* (-0.40)
**Fz**	<0.01* (0.28)	<0.01* (-0.41)	<0.01* (-0.55)
**F4**	0.33 (0.32)	<0.01* (-0.53)	<0.01* (-0.48)
**F7**	<0.01* (-0.34)	<0.01* (-0.40)	<0.01* (-0.56)
**F8**	<0.01* (-0.34)	<0.01* (-0.50)	<0.01* (-0.43)

**Fig 2:**
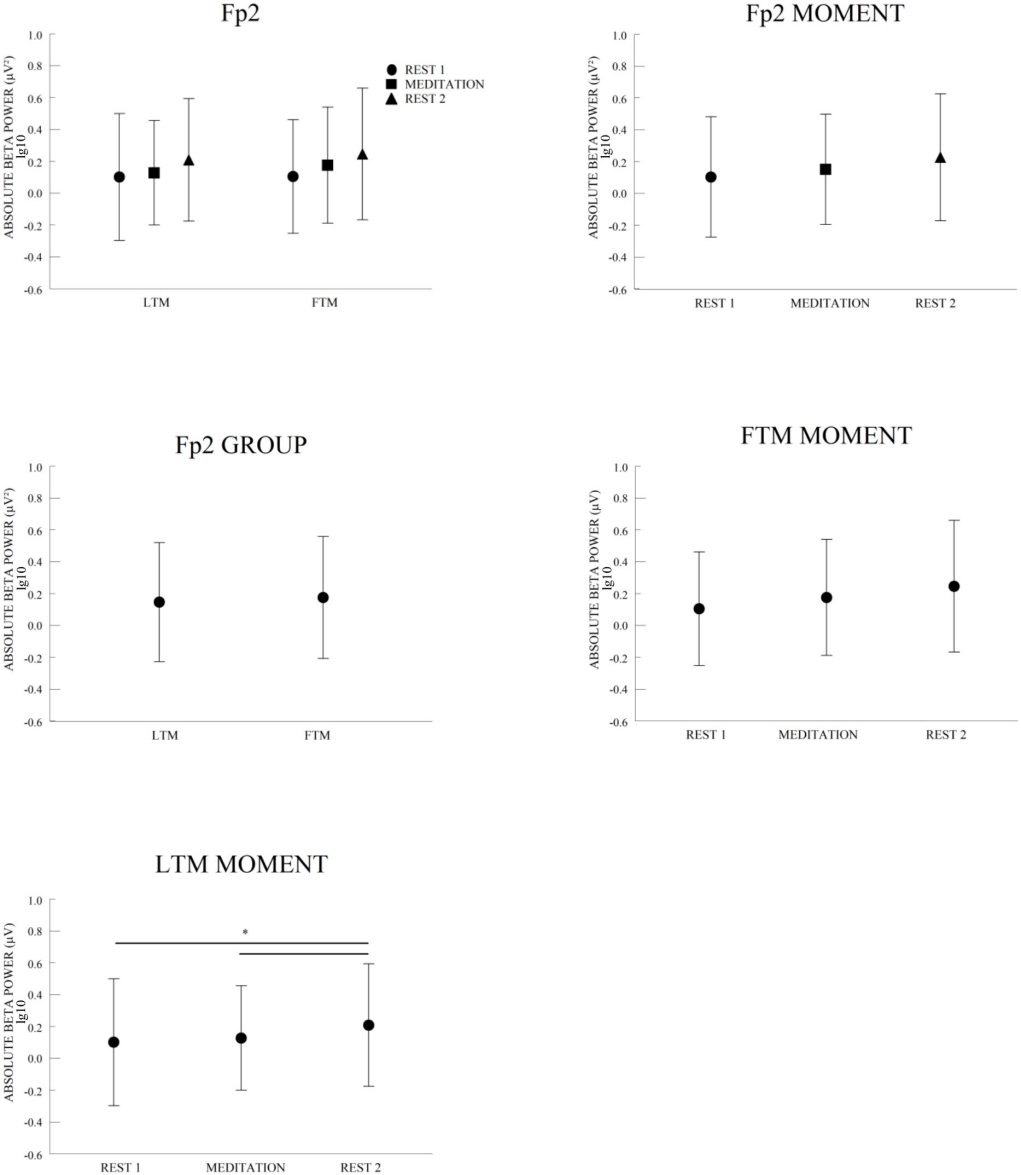
A 2 × 3 ANOVA design showed a main effect for group and moment for Fp2 electrode. a) Comparisons of LTM and FTM absolute beta power (logged mean ± SD) (log10 transformed) showed significant differences between groups (p = 0.02); b) Comparisons of rest 1, meditation and rest 2 absolute beta power (logged mean ± SD) (log10 transformed) showed a significant increase for rest 1 to meditation (p = 0.01), meditation to rest 2 (p<0.01) and rest 1 to rest 2 (p<0.01).

**Fig 3:**
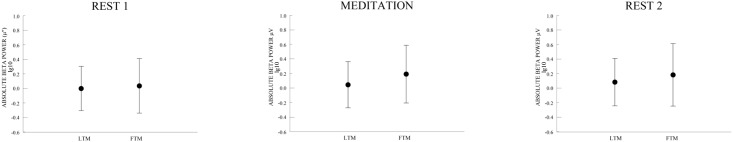
A t-test showed a significant difference for frontal beta activity in the rest 1 (p<0.01), meditation (p<0.01) and rest 2 (p<0.01) when compared LTM to FTM. All frontal electrodes are included (Fp1, Fp2, F3, Fz, F4, F7 and F8) and separated for each moment.

We found a main effect for group and moment for the Fp2 electrode. A main effect for group with a lower absolute beta power was found for the LTM, when compared to the FTM. A main effect for moment with a lower absolute beta power was found at rest 1, rather than for meditation and rest 2; also beta was lower at meditation than at rest 2 (Fig 2). To further explore the Fp2 result, we applied a one-way ANOVA for each group separately. We did not find differences between rest1-meditation, only for LTM (Fig 1 and Table 1). We found significant differences between groups for all frontal electrodes together (Fp1, Fp2, F3, Fz, F4, F7 and F8) using a t-test for each moment (p<0.01) (Fig 3).

## Discussion

The aim of this study was to clarify the neurophysiology of absolute beta power in the frontal area comparing FTM and LTM performing OM meditation. We hypothesized that attentional regulation promotes an altered frontal beta modulation[32–34]. We observed that the maintenance of attention in the present moment produced lower frontal beta power in LTM when compared to FTM (Fig 3), representing a specific meditative trait. Our preliminary results indicate a beta power increase in the frontal area during meditation for both groups. The findings are supported by the previous studies relating beta activity and attentional state [35–37,24,38]. Therefore, some studies have shown different understanding about OM mechanisms, as discussed below.

Frontal beta rhythm is prevalent during attentional activity. However, its role in the meditative state remains unclear. Beta is a low-mid frequency range rhythm that is detected when subjects are alert and in an attentive state [5,35], and also reflects the oscillation of the anticipatory processes in the motor system [36,39]. Moreover, this process seems to be different in OM. Previous studies demonstrated that OM improves self-regulation and stress management by allowing individuals effortless attention processing [40–42].

The literature has shown that the intensity and life-time experience of meditation practice contribute to the progression of the mental training [32,43,44]. Moreover, previous studies have shown that OM practice improves the maintenance of attention depending on the time of training and may contribute to the efficiency of the cognitive processes such as executive processing (i.e. working memory)[34]. Likewise, LTM are able to modulate emotion and cognition through the bottom-up pathways without the main influence of the prefrontal cortex[33].

The opposite pattern is noted with FTM, showing a high cognitive control to modulate attention and perception, suggesting a top-down regulation[32,33]. In this case, it is expected that the FTM present higher beta power. Our results are in agreement with this hypothesis. We found a similar beta pattern for both groups at all the moments investigated (rest 1, meditation, rest 2). However, we observed that FTM presented a higher beta band during meditation when compared to LTM for all electrodes observed, except for the right prefrontal cortex (i.e., Fp2), as showed in Fig 1. In other words, we suggest that FTM`s higher beta in the frontal cortex occurs due to the fact that this group exerts more effort to maintain the attention fixed on a specific thought or sensation, also called “object” (i.e. breath, body sensation); this is seen in FTM more than in LTM, who are already better trained to sustain the OM practice[19].

The OM practice provides a dynamic flow of attention different from focused attention[45]. Some mindfulness body scan studies have shown a misperception decrease and a sensitivity increase[46–48] in case of distress deriving from unpleasant body sensations, such as pain sensitization[49,50]. Recently, Engel and Fries (2010)[36] demonstrated that top-down and bottom-up frontal beta activities are associated with expectancy of the following event, thus also manifesting attentional activity[51].

Although we did not measure the level of expectancy of our subjects, we hypothesized that the beta power increased in FTM was also associated with the level of expectancy and low flexibility of sustained attention without focus on a specific object. In this context, the differences between the groups are associated with the meditator´s ability to modulate sensation and perception strategies [32]. The OM meditative state depends on a specific mental training which is optimized by the experience and/or time of practice, and it is only generated arousal and motivation [51].

Other recent studies have implicated different pathways to explain lower frontal activity during the attentional process, observing that the prefrontal cortex does not modulate attention processing during the OM practice. The discussion is based on the bottom-up processing during OM meditative state. This meditative practice, often viewed as an emotional regulation strategy, has been associated with a lower frontal cortex activity [32,52]. At the same time hyperactivity occurs in the prefrontal cortex when related to an altered state working memory, executive attention, emotional reappraisal and cognitive monitoring[32,33,53].

Furthermore, this increased activity is also associated with the narrative focus, occurring mainly in the medial prefrontal cortex. The typical narrative focus (e.g. discursive thought) impairs attention-task performance, involving mental elaboration and evocation and overloading working memory processies [54]. Examining the interaction in Fp1, we observed that for FTM the rest 1 is different from the meditation moment; and we did not find this difference for the LTM (Fig 1 and Table 1). Our results also demonstrated that lower beta power found for LTM is related to effortless attention, which is not found for FTM. This finding suggest that experienced meditators are trained to maintain the attention state even before starting the meditation; which suggests that the meditation training regularity improves attention. The LTM medial prefrontal cortex (Fz) showed higher beta power at rest 1, due to the maintenance of the self-referential mental processing even when not meditating[51]. This pattern was different during meditation on account of a lower activity of the LTM supporting the effortless hypothesis. In both situations, increased beta power was observed during meditation, but the FTM showed higher power than LTM.

In conclusion, LTM have a particular trait, which differentiates them when compared to FTM. Lower frontal beta activity is associated with the particularity of OM being related to bottom-up pathways. On the other hand, an increased activity in the frontal area has been found when attention was focused on a specific object, used as an attentive state maintenance tool by FTM more than by LTM. We hypothesize that this pattern associated with LTM improved their attentive state, while maintaining low cognitive demands. Due to the enhanced learning, related both to time and intensity of practice, it may be a promising biomarker to differentiate experienced mindfulness practitioners in further studies.
